# Identification of a Six-Immune-Related Long Non-coding RNA Signature for Predicting Survival and Immune Infiltrating Status in Breast Cancer

**DOI:** 10.3389/fgene.2020.00680

**Published:** 2020-07-07

**Authors:** Zheng Li, Yaming Li, Xiaolong Wang, Qifeng Yang

**Affiliations:** ^1^Department of Breast Surgery, General Surgery, Qilu Hospital of Shandong University, Jinan, China; ^2^Pathology Tissue Bank, Qilu Hospital of Shandong University, Jinan, China

**Keywords:** breast cancer, immune infiltration, long non-coding RNA, gene expression omnibus, the cancer genome atlas, prognostic signature

## Abstract

Long non-coding RNAs (lncRNAs) play critical roles in tumor immunity; however, the functional roles of immune-related lncRNAs in breast cancer (BC) remain elusive. To further explore the immune−related lncRNAs in BC, whole genomic expression data and corresponding clinical information were obtained from multiple BC datasets. Based on correlation with the immune-related genes within the training set, we screened out the most promising immune-related lncRNAs. Subsequently, Lasso penalized Cox regression analysis followed by stepwise multivariate Cox regression analysis identified six survival-related lncRNAs (AC116366.1, AC244502.1, AC100810.1, MIAT, AC093297.2, and AL356417.2) and constructed a prognostic signature. The cohorts in the high−risk group had significantly poor survival time compared to those in the low−risk group. In addition, a nomogram integrated with clinical features and the prognostic signature was developed on the basis of the training set. Importantly, all the findings had a similar performance in three validated datasets. In the following studies, our integrative analyses indicated that the infiltration of CD8-positive (CD8) T cells associated with a good prognosis was strikingly activated in the low−risk group. To further provide an interpretation of biological mechanisms for the prognostic signature, we performed weighted gene co−expression network analysis (WGCNA) followed by KEGG pathway-enrichment analysis. Our results showed that the antigen presentation pathway involved in protein processing in endoplasmic reticulum and antigen processing and presentation was markedly altered in the high-risk group, which might promote tumor immune evasion and associate with poor clinical outcomes in BC patients with high risk scores. In conclusion, we aimed to take advantage of bioinformatics analyses to explore immune−related lncRNAs, which could function as prognostic indicators and promising therapeutic targets for BC patients.

## Introduction

Breast cancer (BC) is one of the most frequent malignant tumors and a leading cause of cancer death among females (Siegel et al., 2019). Although comprehensive treatments including surgery, radiotherapy, and chemotherapy have been well supplied, the prognosis of BC patients is not satisfactory (Schwartz and Erban, 2017; Emens, 2018). Therefore, it is urgent to have an insight into novel prognostic and diagnostic markers to further develop novel therapeutic approaches for BC patients.

The tumor microenvironment (TME) is a complicated system, consisting of not only tumor cells but also tumor-associated normal epithelial and stromal cells, immune cells, and vascular cells (Junttila and de Sauvage, 2013). Emerging evidence has identified that dysfunctional immune status in TME is a hallmark of cancers (Yu et al., 2018). Infiltrating immune cells in TME play critical roles in the initiation and progression of cancer, and positively or negatively influence patient prognosis, which is primarily due to tumor heterogeneity and distinct populations of tumor-infiltrating immune cells (Talmadge et al., 2007). The infiltration of regulatory T (Treg) cells inhibits endogenous immune responses against tumors and correlates with poor prognosis (Joshi et al., 2015), whereas dendritic cell (DC) infiltration is typically associated with a positive clinical outcome associated with their capacity to initiate and regulate both innate and adaptive immunity (Saxena and Bhardwaj, 2018). Myeloid-derived suppressor cells (MDSCs) could prevent cytotoxic T lymphocytes (CTLs) from binding to the peptide–MHC complex and therefore inhibit antitumor activity (Nagaraj et al., 2007). In contrast, infiltration of CD8-positive cytotoxic T lymphocytes (CD8 T cells) into solid tumors is associated with good prognosis in various types of cancer, including BC (Jiang and Shapiro, 2014; Li et al., 2019). CD4-positive T cells, namely, T follicular helper (TFH) cells, have two predominant cell subtypes, Th1 and Th2. In general, infiltration of TH1 cells predicts a positive outcome, whereas TH2 cells predict a negative outcome (Giraldo et al., 2014; Pradhan et al., 2014). Natural killer (NK) cells, lymphocytes of the innate immune system, are essential for defense against infectious pathogens and nascent malignant cells. Classically, the CD56dim NK cell subset is considered to mediate antitumor responses, whereas the CD56bright NK cell subset is involved in immunomodulation. Nevertheless, current studies demonstrated that brief priming with IL-15 strikingly enhanced the antitumor response of CD56 bright NK cells (Wagner et al., 2017). Given that clinical outcomes of cancer patients were frequently associated with the infiltration of immune cells in TME, current diagnostic and therapeutic strategies are mainly focused on the analysis and manipulation of infiltrating immune cellular populations (Talmadge et al., 2007; Tamborero et al., 2018).

Long non-coding RNAs (lncRNAs), longer than 200 nucleotides, play crucial roles in the occurrence and progression of cancers (Iyer et al., 2015). Previous studies have shown that lncRNAs could interact with other biomolecules, such as proteins, regulatory DNA regions, and miRNAs, and therefore regulate gene expression at multiple levels, including epigenetic, transcriptional, and post-transcriptional (Wu et al., 2018). Recently, mounting studies found that the lncRNAs also play a pivotal role in cancer immunity (Hu et al., 2013; Heward and Lindsay, 2014). For instance, lincRNA-Cox2, proximal to the prostaglandin-endoperoxide synthase 2 (Ptgs2/Cox2) gene, regulated both the activation and repression of diverse types of immune genes (Carpenter et al., 2013; Hu et al., 2016). LncRNA FENDRR had an impact on the immune escape of hepatocellular carcinoma cells through interaction with miR-423-5p and GADD45B (Yu et al., 2019). Another research revealed that lncRNA NKILA overexpression in tumor-specific CTLs and TH1 promoted their apoptosis and contributed to shorter survival time in patients with BC or lung cancer (Huang et al., 2018). However, the immune−related lncRNAs associated with BC progression remain poorly understood.

In this study, we integrated bioinformatics analyses and took advantages of lncRNA expression profiles to explore novel prognostic marks and therapeutic targets for BC patients.

## Materials and Methods

### Sample Datasets and Data Processing

The GSE20685 dataset was downloaded from the GENE EXPRESSION OMNIBUS (GEO) which contains 327 BC cases. This dataset was based on the GPL570 platform (Affymetrix Human Genome U133 Plus 2.0 Array). Meanwhile, patients’ clinical information was also obtained from the GEO database, including age, overall survival time (OS), survival status, and clinical stage. We re-annotated probe sets of Affymetrix Hg-U133 Plus 2.0 array by mapping all probes to the human genome (GRCh37) using SeqMap (Jiang and Wong, 2008). In the analysis, we ensure that those probes mapped to the genome were unique with no mismatch, and all probes mapped to pseudogene transcripts were removed. The analysis procedure was conducted by the previous study (Du et al., 2013).

### Construction and Validation of Immune-Related LncRNAs Prognostic Signature

Firstly, the immune−related genes were extracted from immune system process and immune response gene sets sourced from the Molecular Signatures Database (MSigDB,^1^) (Liberzon et al., 2015). Then, a cohort of immune−related lncRNAs was identified according to Pearson correlation analysis between immune genes and lncRNA expression level in BC samples (|*r*| > 0.5, *p* < 0.001). Next, the univariate Cox regression analysis and Kaplan–Meier method were separately performed to sort out survival−related lncRNAs with significant prognostic value (*p* < 0.05) on the basis of the training set. Following these two independent analyses, the overlapping lncRNAs with significant difference (*p* < 0.05) were sent for further analyses. Finally, Lasso penalized Cox regression analysis followed by stepwise multivariate Cox regression analysis identified the most promising survival-related lncRNAs and constructed a prognostic signature.

The enrichment scores (ES) of immune genes associated with the prognostic signature were calculated using the Single-sample Gene Set Enrichment Analysis (ssGSEA) in Gene Set Variation Analysis (GSVA) package (Hanzelmann et al., 2013), and then the immune signature based on enrichment scores was developed. The Pearson correlation analysis was employed to evaluate the relationship between the prognostic signature and the immune signature. Subsequently, random forest (RF) and logistic regression (LR), two well machine learning algorithms, were applied to evaluate the performance of the six-lncRNA signature for stratifying the immune status based on the median enrichment score of immune signature according to the area under the ROC curve (AUC) through five-fold cross validation (Han et al., 2014).

### Risk Score Construction

To confirm the reliability of the detected immune−related lncRNAs, the risk score was designed to establish a unitive signature for the prognostic analysis, and the coefficient for each lncRNA was obtained through the multivariate cox regression following bidirectional stepwise selection. A risk score formula was established as follows:

$$\sum_{\text{i}}\mathrm{Coefficient}\left({\mathrm{IncRNA}}_{\text{i}}\right)\times \mathrm{Expression}\left({\mathrm{IncRNA}}_{\text{i}}\right)$$

Risk score = (−0.468 × expression of AC116366.1) + (−0.260 × expression of AC244502.1) + (−0.279 × expression of AC100810.1) + (−0.162 × expression of MIAT) + (−0.312 × expression of AC093297.2) + (0.173 × expression of AL356417.2).

### Nomogram Construction

A nomogram integrated clinical features and the six-lncRNA signature was constructed using the “rms” R package. Calibration curve were performed to assess the predictive accuracy of the nomogram. The predicted outcomes and actual observed outcomes of the nomogram were presented in the calibrate curve, and the 45° line represents the best prediction.

### Gene Set Enrichment Analysis

To further explore the relationship between immune-related gene sets and the six-lncRNA signature, GSEA was performed with the JAVA program. Genes were ranked on the basis of differential significance between the high- and low- risk groups, which were stratified via the median risk score in BC patients. After performing 1000 permutations, gene sets enrichment with nominal *p* < 0.05 and FDR < 0.25 were considered significant.

### Weighted Gene Co-expression Network Analysis (WGCNA)

The WGCNA package (Langfelder and Horvath, 2008) was applied for further analysis on the basis of the training set, and the analysis procedure was conducted as previously described (Giulietti et al., 2016). In brief, two weighted gene co-expression networks were constructed via the WGCNA package according to quartile of risk scores, with the bottom group serving as the reference network and the top group serving as the test network. The conservation of gene pairs between two networks was evaluated by module preservation function from WGCNA. In order to make analysis more accurate, 1000 times of permutation were performed. Subsequently, median rank was used to confirm non-preservative modules and *Z*-summary score (*Z*-score) was applied to evaluate the significance of non-preservative modules. Note that *Z*-scores greater than 10 indicate a high preservation and *Z*-scores less than 10 indicate a weak preservation. Finally, the genes in non-preservation modules were enrolled to send for DAVID tool (version 6.8) (Huang et al., 2007) to map these hub genes to KEGG pathways. Enriched KEGG pathways with *p*-values < 0.05 were extracted, and then the enrichment results were visualized by ggraph package.

### External Data Validation

To further validate predictive capability of the six-lncRNA signature, we enrolled three independent datasets, including GSE21653, GSE88770, and TCGA with a total of 245, 117, and 888 cases, respectively. GSE21653 and GSE88770 were based on the platform GPL570 (Affymetrix Human Genome U133 Plus 2.0 Array). The TCGA dataset (level3, raw count) was obtained from The Cancer Genome Atlas (TCGA) via the TCGAbiolinks package. Patients without complete information in training and validation sets had been excluded from this study. The inclusion patient ids are listed in Supplementary Table S1.

### Statistical Analysis

In Kaplan–Meier survival analysis, the “surv_cutpoint” function in survminer package was applied to determine the optimal cutoff value of risk scores. Note that the amount of samples in any group could not be less than 30% of the total samples. According to the optimal cutoff value, BC patients were divided into high− and low−risk groups to perform survival analysis. The time−dependent receiver operating characteristic (ROC) analysis was performed to investigate the prognostic accuracy of the six−lncRNA signature using the survivalROC package. After the ssGSEA method calculated the enrichment scores of 24 immune cell types (Bindea et al., 2013), Pearson correlation analysis was performed to evaluate the relationship between risk scores and infiltrating immune cells. Logistic regression analysis was carried out to evaluate whether combination of the six lncRNAs have higher predictive accuracy for infiltrating status of CD8 *T*-cells than each predictor alone. Differences with *p* < 0.05 were considered significant.

## Results

### Construction of Immune-Related LncRNAs Prognostic Signature

**FIGURE 1:**
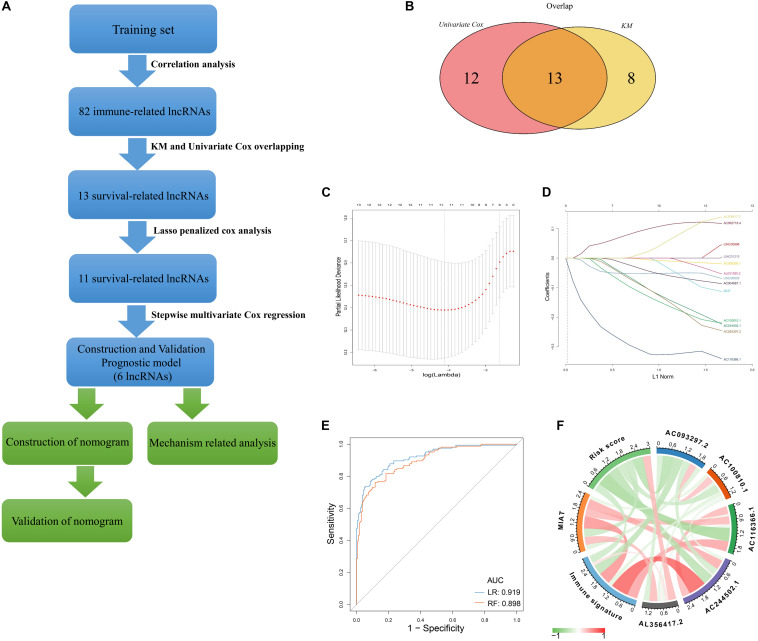
Construction of Immune–related LncRNAs Prognostic Signature **(A)** Flow chart indicating the analysis procedure. **(B)** Survival analysis was separately performed by univariate Cox regression analysis and Kaplan–Meier method, of which 13 survival-related lncRNAs were overlapped. **(C)** The selection of the tuning parameter via 10 times cross–validation in the Lasso model. **(D)** Lasso coefficient profiles of 13 lncRNAs. **(E)** The predictive efficacy of the six-lncRNA signature in classification of immune signature. RF: random forest; LR: logistic regression. **(F)** The correlation identified by Pearson correlation analysis among immune signature, six-lncRNA signature, and six lncRNAs.

A flow chart described our analysis procedure was presented in Figure 1A. In our study, 327 samples sourced from GSE20685 served as the training set. A total of 313 immune−related genes were obtained from the Molecular Signatures Database, which were associated with immune response and immune system process (Supplementary Table S2). Next, 82 immune−related lncRNAs were extracted using Pearson correlation analysis (|*r*| > 0.5, *p* < 0.001) (Supplementary Table S3). Univariate Cox regression analysis and Kaplan–Meier method was separately performed to identify survival-related lncRNAs (*p* < 0.05), of which 13 were overlapped (Figure 1B and Supplementary Table S4). After primary filtration, Lasso penalized Cox regression analysis following stepwise multivariate Cox regression analysis was carried out to sort out six survival-related lncRNAs and developed a prognostic signature (Figures 1C,D; Supplementary Table S5). Moreover, a regulatory network associated with 6 lncRNAs and 86 signature-related immune genes was established and visualized using Cytoscape3.7.1 (Supplementary Figure S1).

Based on 86 model-related immune genes (Supplementary Table S6), we constructed an immune signature via the ssGSEA method. The enrichment scores of the immune signature are listed in Supplementary Table S7. Subsequently, random forest (RF) and logistic regression (LR) revealed that the prognostic signature could significantly stratify two immune subgroups on the basis of the median enrichment score (LR, AUC score = 0.919, RF, AUC score = 0.898) (Figure 1E). Moreover, the interplay among immune signature, prognostic signature, and six lncRNAs was evaluated by Pearson correlation analysis (Figure 1F; Supplementary Table S8). We observed that the six-lncRNA signature was negatively associated with the immune signature (*r* = -0.342, *p* = 1.99E-10). In the prognostic signature, AC116366.1, AC244502.1, AC100810.1, MIAT, and AC093297.2 acted as the protective factors with an HR value less than 1, and AL356417.2 acted as the deleterious factor with an HR value greater than 1 (Table 1).

**TABLE 1:** Six immune-related lncRNAs of the prognostic signature.

		Pearson correlation analysis		Univariate Cox regression analysis			
lncRNAs	Ensemble_ID	*R*	*p*-value	HR	HR.95L	HR.95H	*p*-value
AC116366.1	ENSG00000234290.2	0.462	1.12E−18	0.427	0.280	0.649	6.76E−5
AC244502.1	ENSG00000251002.6	0.786	6.59E−70	0.777	0.660	0.915	0.0025
AC100810.1	ENSG00000253982.1	–0.195	0.0004	0.705	0.563	0.882	0.0023
MIAT	ENSG00000225783.5	0.540	3.54E−26	0.853	0.746	0.975	0.0202
AC093297.2	ENSG00000272335.1	–0.446	2.04E−17	0.757	0.628	0.912	0.0034
AL356417.2	ENSG00000233682.3	0.140	0.0112	1.319	1.079	1.613	0.0070

*R: Correlation of six lncRNAs and the immune signature.*

### Primary Evaluation of the Six-LncRNA Signature in the Training Set

**FIGURE 2:**
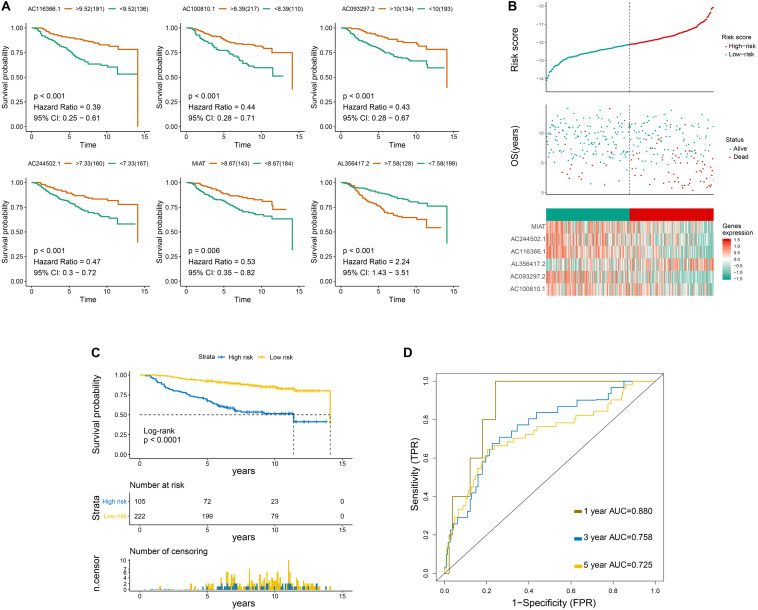
Primary evaluation of the six-LncRNA Signature in the Training Set. **(A)** Overall survival curves of six lncRNAs. **(B)** From top to bottom are the risk score, survival status distribution, and six-lncRNA expression level of each patient. **(C)** Overall survival curves of the prognostic signature, in which the blue line represents the high-risk subgroup and the yellow line represents the low-risk subgroup. **(D)** The 1-, 3-, and 5-year survival receiver operating characteristic curves.

Overall survival curves of six immune-related lncRNAs are shown in Figure 2A. Risk scores, survival status, OS period, and six-lncRNA expression level of each patient are presented in Figure 2B. Moreover, patients in the low−risk group showed increased survival time in contrast to those in the high−risk group (Figure 2C). Univariate following multivariate Cox regression analysis was conducted to identify that N-stage and the six-lncRNA signature functioned as independent prognostic factors (Supplementary Figures S2, S3). The predictive accuracy of the six-lncRNA signature was assessed by time−dependent ROC curves at 1, 3, and 5 years on the basis of AUC scores. The AUC scores at 1, 3, and 5 years were 0.88, 0.758, and 0.725, respectively, which indicated that the prognostic signature had a moderate sensitivity and specificity in the training set (Figure 2D).

### Further Evaluation of the Six-LncRNA Signature in Validation Sets

**FIGURE 3:**
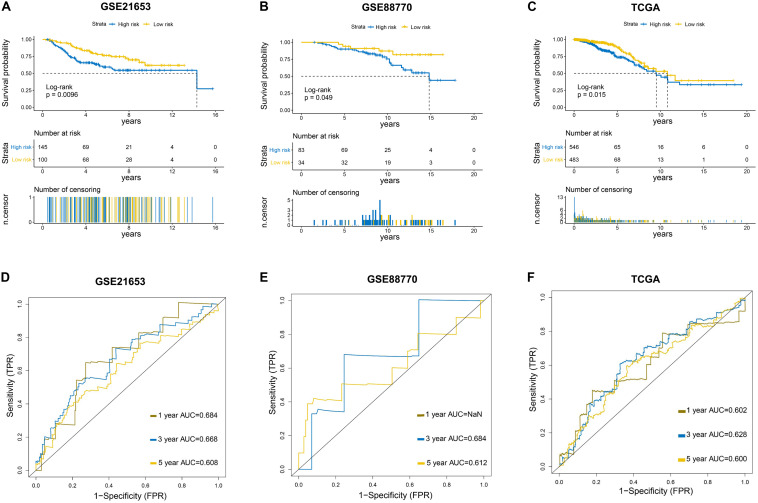
Further evaluation of the six-LncRNA Signature in Validation Sets. **(A–C)** Survival curves of the prognostic signature in three validation sets. GSE21653: Disease-free survival (DFS) curve. GSE88770 and TCGA: Overall survival (OS) curve. **(D–F)** Survival receiver operating characteristic curves in three validation sets.

To further evaluate the predictive efficacy of the six−lncRNA signature, three independent external datasets (GSE21653, GSE88770, and TCGA) were used to validate our findings. Kaplan–Meier analysis showed that patients in the low-risk group had evidently longer survival time than those in the high-risk group in three validation sets (Figures 3A–C), which was consistent with the results from the training set. The ROC curves at 1, 3, and 5 years indicated that the signature also had a good predictive performance in validation sets (Figures 3D–F).

### Construction and Evaluation of Nomogram

**FIGURE 4:**
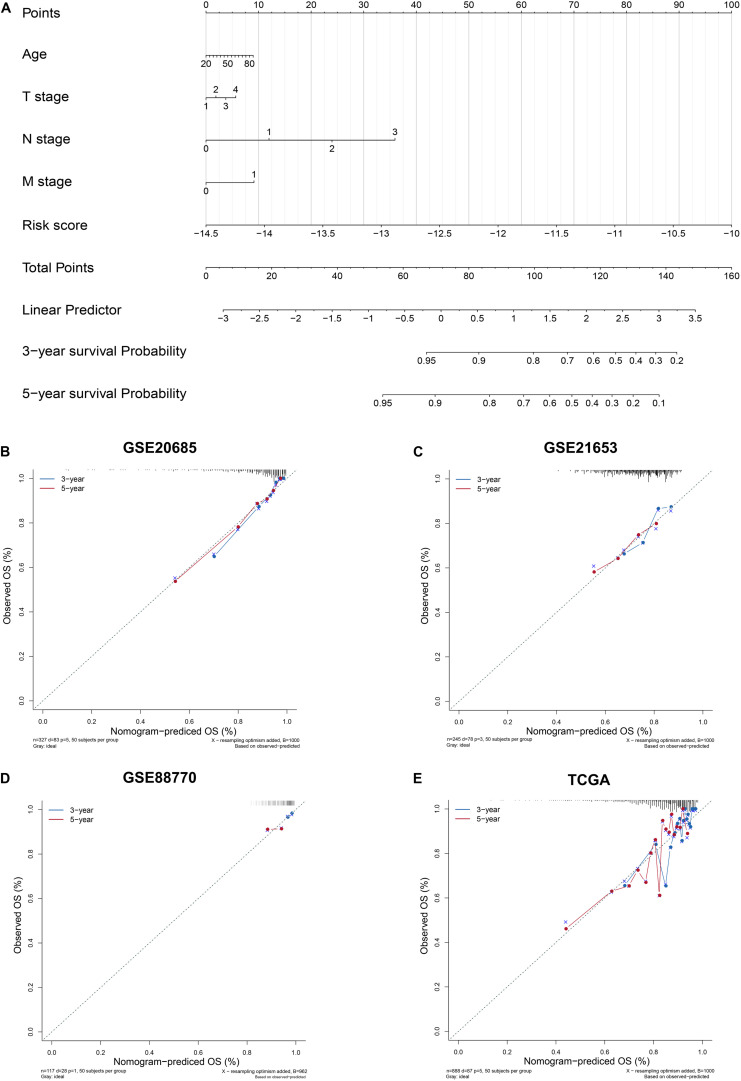
Construction and Evaluation of Nomogram. **(A)** Nomogram to predict survival probability. **(B–E)** Three- and 5-year nomogram calibration curves in training and validation sets. The training set: GSE20685. The validation sets: GSE21653, GSE88770, and TCGA.

To enhance the predictive accuracy, four clinical features and the six-lncRNA signature were integrated and a nomogram was constructed on the basis of the training set (Figure 4A). The calibration curves presented a good agreement between prediction by nomogram and actual observation for predicting survival probability at 3 and 5 years in training and validation datasets (Figures 4B–E).

### Immune Status Analysis for High- and Low-Risk Groups

**FIGURE 5:**
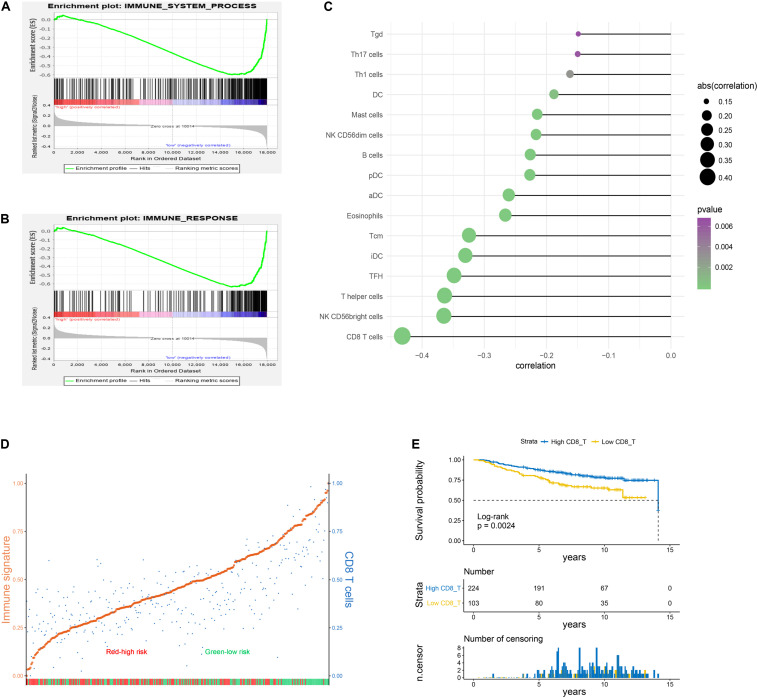
Immune status analysis for high– and low–risk groups. **(A,B)** Gene set enrichment analysis (GSEA) indicated a significant enrichment of immune–related phenotype in the low–risk group. **(C)** Pearson correlation analysis between risk scores of the six-lncRNA signature and enrichment scores of the immune cells. The results with *p*-value < 0.05 were extracted. **(D)** Enrichment scores of immune signature and CD8 T cells in high- and low-risk cohorts. The patients were divided into two subgroups on the basis of the median risk score. The enrichment score was scaled to be between 0 and 1 in the plot. Orange: immune signature; blue: CD8 T cells; red: high-risk patients; green: low-risk patients. **(E)** Kaplan–Meier curve associated with CD8 T cells.

In the following study, GSEA was applied to show that immune system process and immune response gene sets were significantly enriched in the low−risk group (Figures 5A,B). The ssGSEA followed by Pearson correlation analysis was conducted to validate the relationships between risk scores and infiltration immune cells. As presented in Figure 5C, risk scores had the strongest correlation with CD8 T cells compared to other types of immune cells. Based on the median risk score, BC patients in the training set were stratified into two subgroups, high- and low-risk groups. High-risk cohorts displayed lower enriched levels of the immune signature and CD8 T cells compared to low-risk cohorts (Figure 5D). Meanwhile, the immune signature was strikingly positively related with CD8 T cells on the basis of Pearson correlation analysis (*r* = 0.72, *p* = 1.54E-53; Supplementary Figure S4). Survival curve revealed that infiltration of CD8 T cells was associated with a favorable prognosis in BC patients (Figure 5E).

**FIGURE 6:**
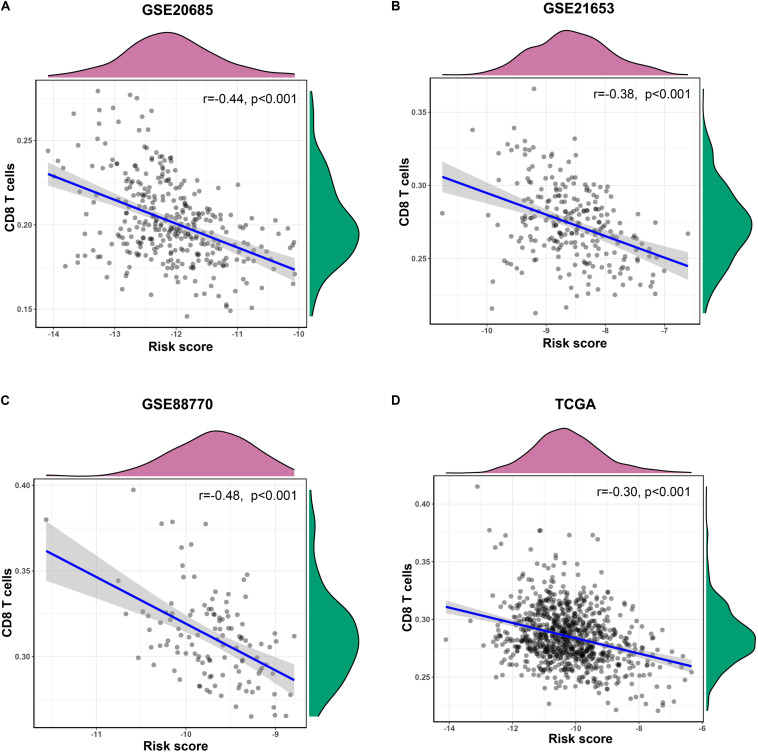
The correlation of the six-lncRNA signature and CD8 T cells. **(A–D)** Pearson correlation analysis was conducted to identify the relationship between risk scores of the prognostic signature and enrichment scores of CD8 T cells in training and validation sets.

**FIGURE 7:**
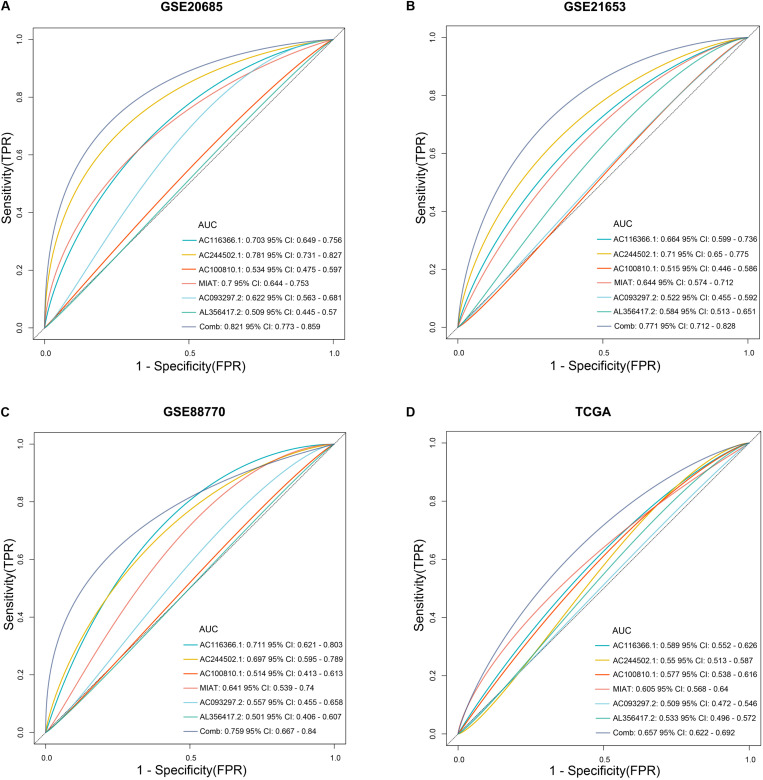
The predictive efficacy of the six-lncRNA signature for infiltration of CD8 T cells. **(A–D)** Logistic regression was conducted to evaluate the predictive efficacy of the combination of the six lncRNAs and each lncRNA alone on the basis of the AUC value in training and validation sets.

In training and validation sets, risk scores were negatively related with enrichment scores of CD8 T cells on the basis of Pearson correlation analysis (Figures 6A,D). To further identify predictive efficacy of the signature for infiltration of CD8 T cells, the patients of training and validation sets were divided into high- and low-infiltration groups according to the median enrichment score of CD8 T cells. Logistic regression analysis showed that the combination of six lncRNAs had higher predictive efficacy than each predictor alone (Figures 7A–D).

### Construction of Weighted Gene Co-expression Network and Identification of Key Modules

**FIGURE 8:**
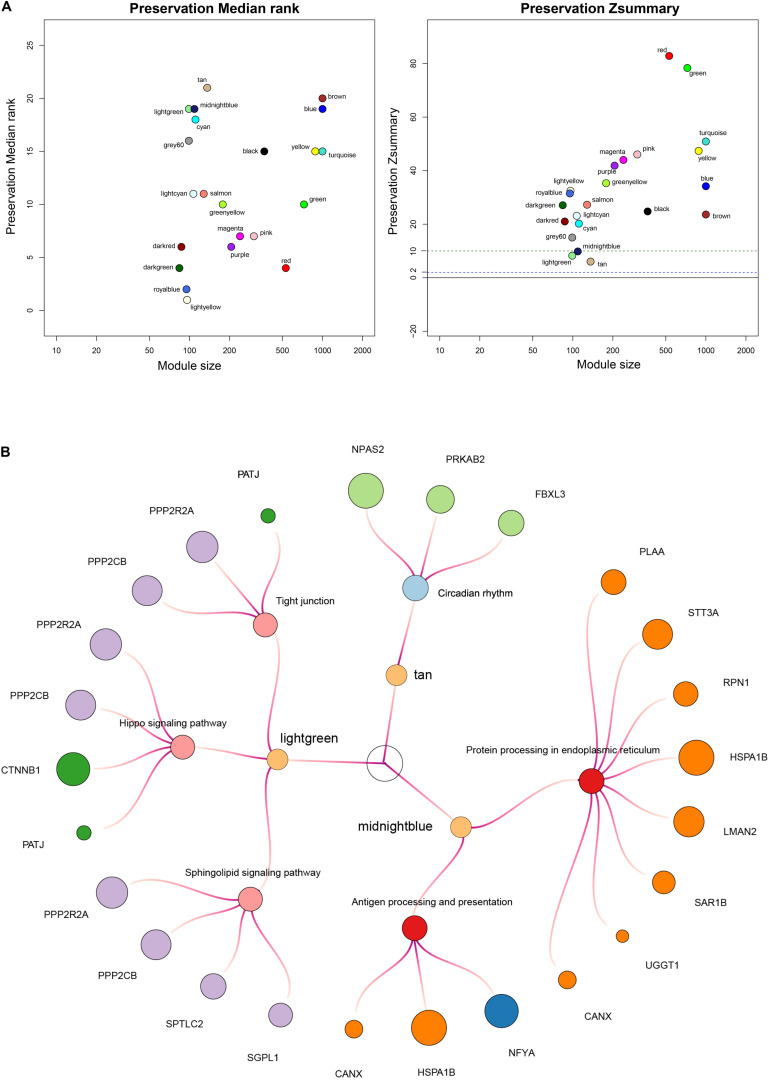
Construction of weighted co–expression network and identification of key modules. **(A)** Each module is represented by its color-code and name. Left plot shows the preservation median rank, which tends to be independent from module size with high median ranks indicating low preservation. Right plot shows *Z*-score value. The green dotted line indicates that *Z*-score is equal to 10. *Z*-score < 10 represents weak preservation. **(B)** Enriched KEGG pathways with *p*-values < 0.05 were presented. KME value (eigengene connectivity) of module internal hub genes was indicated by dots’ size, respectively.

In the following study, we applied the WGCNA method to identify non-preservation modules, whose characteristics could distinguish the test network from the reference network. Firstly, we confirmed a soft threshold power value of 16 as best optimum index in the high-risk group (Supplementary Figure S5) and 2 as the best optimum index in the low-risk group (Supplementary Figure S6). Next, we identified 6 modules in the high-risk group and 22 modules in the low-risk group except for the gray module via hierarchical clustering. Finally, the module preservation function in the WGCNA package was applied to identify three non-preservation modules, the light green module (*Z*-score = 8.2), the midnight blue module (*Z*-score = 9.8) and the tan module (*Z*-score = 6.0) (Figure 8A).

To further investigate the biological mechanisms of non-preservation modules, we mapped internal genes of non-preservation modules to KEGG pathways using the DAVID tool (version 6.8) (Huang et al., 2007). Enriched KEGG pathways with *p* values < 0.05 were extracted. KEGG pathway-enrichment analysis demonstrated that the midnight blue module was mainly involved in protein processing in endoplasmic reticulum and antigen processing and presentation; the light green module was mainly involved in sphingolipid signaling pathway, hippo signaling pathway, and tight junction; and the tan module was mainly involved in circadian rhythm (Figure 8B). In summary, the midnight blue module could be a crucial module, whose disorganized functions affected immune cell infiltration and contribute to shorter survival time in patients with high risk scores.

## Discussion

Infiltration of immune cells in TME has dual roles on cancer progression and patient prognosis, and deciphering their complex interplay is of seminal importance for exploring novel prognostic markers and therapeutic targets in cancer patients (Fridman et al., 2011; Klemm and Joyce, 2015). LncRNAs have been reported to function as key regulators in immune response, inflammation, and distinct immune cell types (Das et al., 2018; Li et al., 2018; Wang et al., 2018; Xu and Cao, 2019), and play crucial roles in cancer malignant progression (Bartonicek et al., 2016; Jiang et al., 2016; Schmitt and Chang, 2016). Therefore, insight into immune-related lncRNAs could bring profound effects on cancer-related therapeutics and survival prognosis prediction (Chen et al., 2017).

Since the lncRNA expression level and biological functions were markedly associated with immune-related genes (Roux et al., 2017), we employed immune-associated gene sets to screen out highly immune-related lncRNAs by co-expression analysis. Subsequently, a series of bioinformatics analyses were integrated to identify a six-immune-related lncRNA signature, which could well stratify patients into favorable or unfavorable prognoses. Moreover, the six-lncRNA signature was considered as an independent risk factor by multivariate Cox regression analysis. To further improve the predictive accuracy, a nomogram consisting of age, TMN stage, and the six-lncRNA signature was constructed. A good performance of the nomogram was identified by the calibration curve. Meanwhile, our findings were validated in multiple BC datasets. Additionally, functional annotation conducted by GSEA revealed that the immune-related gene sets, the immune system process, and immune response were significantly activated in the low-risk group.

Previous studies have shown that different types of infiltrating immune cells have distinct effects on tumor progression (Fridman et al., 2012; Bindea et al., 2013). In order to investigate the association between infiltration immune cells and the lncRNAs signature, the ssGSEA method was used to calculate enrichment scores of 24 immune cell types (Bindea et al., 2013), and then Pearson correlation analysis was conducted to evaluate their correlations. Our integrated analyses indicated that risk scores were significantly associated with the enrichment level of CD8 T cells. In addition, the combination of six lncRNAs had higher prognostic accuracy for CD8 T cell infiltrating status than each predictor alone, indicating that the signature could provide promising immunotherapeutic targets for the treatment of BC. CD8 T cells function as a tumor suppressor and play crucial roles in cancer progression (Barry and Bleackley, 2002; Vesely et al., 2011; Jansen et al., 2019). In BC, previous studies have demonstrated that CD8 T cells are the primary effector immune cells and associated with favorable clinical outcomes of BC patients (Mahmoud et al., 2011). Taken together, dysregulation of CD8 T cells might be a key mechanism associated with a higher mortality of patients in the high-risk group.

To further provide an interpretation of biological mechanisms for the six-lncRNA signature, we performed WGCNA analysis to explore non-preservative modules in the high-risk group compared with the low-risk group. Subsequent KEGG pathway-enrichment analysis demonstrated that the midnight blue module was strongly associated with immune-related pathways mainly involved in protein processing in endoplasmic reticulum and antigen processing and presentation. In the immune system, the antigen presentation pathway plays central roles for the immune surveillance and elimination of malignant transformations (Hu et al., 2019). Nascent malignant cells may reduce antigenicity so that anti-tumor lymphocytes fail to detect transformed cells to escape immune surveillance (Hu et al., 2019). Endoplasmic reticulum (ER) could process and present mutation-derived tumor-related nascent antigens to CD8 T cells, which contribute to tumor suppression (Johnsen et al., 1999; Hu et al., 2019). In our study, the antigen presentation pathway involved in protein processing in endoplasmic reticulum and antigen processing and presentation was disorganized in the high-risk group, which might promote tumor immune evasion and associate with poor clinical outcomes in the patients with high risk scores.

In the study, we integrated bioinformatics analyses to provide a solid theoretical basis for potential value of the six-lncRNA signature. However, in the process of public database mining, some unknown factors are usually difficult to extrapolate. In subsequent studies, we will further demonstrate and improve our findings by more rigorous experimental evidence in immune-related lncRNAs associated with BC.

## Conclusion

In conclusion, we integrated informatics analyses to identify a six immune-related lncRNAs signature. Moreover, CD8 T cell infiltration was significantly activated in the low-risk group, which could contribute to a better prognosis of BC patients. Our findings aim to help improve clinical outcome prediction and provide promising immunotherapeutic targets for BC patients.

## Data Availability Statement

Publicly available datasets were analyzed in this study, these can be found in The Cancer Genome Atlas (http://cancergenome.nih.gov/) and the NCBI Gene Expression Omnibus (https://www.ncbi.nlm.nih.gov/geo/) (GSE20685, GSE21653, and GSE88770).

## Author Contributions

QY conceived and directed the project. ZL designed the study and analyzed the data. ZL and YL wrote the manuscript. XW reviewed the data. All authors have read and approved the final manuscript for publication.

## Conflict of Interest

The authors declare that the research was conducted in the absence of any commercial or financial relationships that could be construed as a potential conflict of interest.

## Footnotes

1 https://www.gsea-msigdb.org/gsea/msigdb/

## Abbreviations

AUC: the area under the ROC curve
BC: breast cancer
CD8 T cells: CD8-positive cytotoxic T lymphocytes
DFS: disease-free survival
FDR: false discovery rate
GEO: the Gene Expression Omnibus
GSEA: gene set enrichment analysis
GSVA: gene set variation analysis
HR: hazard ratio
KEGG: Kyoto Encyclopedia of Genes and Genomes
lncRNAs: long non-coding RNAs
LR: logistic regression analysis
MSigDB: the Molecular Signatures Database
OS: overall survival
RF: random forest
ROC curve: receiver operating characteristic curve
ssGSEA: single sample gene set enrichment analysis
TCGA: The Cancer Genome Atlas
TME: the tumor microenvironment
WGCNA: weighted gene co-expression network analysis.

## References

[B1] Barry M. Bleackley R. C. (2002). Cytotoxic T lymphocytes: all roads lead to death. *Nat. Rev. Immunol.* 2 401–409. 10.1038/nri819 12093006

[B2] Bartonicek N. Maag J. L. Dinger M. E. (2016). Long noncoding RNAs in cancer: mechanisms of action and technological advancements. *Mol. Cancer* 15:43. 10.1186/s12943-016-0530-6 27233618 PMC4884374

[B3] Bindea G. Mlecnik B. Tosolini M. Kirilovsky A. Waldner M. Obenauf A. C. (2013). Spatiotemporal dynamics of intratumoral immune cells reveal the immune landscape in human cancer. *Immunity* 39 782–795. 10.1016/j.immuni.2013.10.003 24138885

[B4] Carpenter S. Aiello D. Atianand M. K. Ricci E. P. Gandhi P. Hall L. L. (2013). A long noncoding RNA mediates both activation and repression of immune response genes. *Science* 341 789–792. 10.1126/science.1240925 23907535 PMC4376668

[B5] Chen Y. G. Satpathy A. T. Chang H. Y. (2017). Gene regulation in the immune system by long noncoding RNAs. *Nat. Immunol.* 18 962–972. 10.1038/ni.3771 28829444 PMC9830650

[B6] Das S. Reddy M. A. Senapati P. Stapleton K. Lanting L. Wang M. (2018). Diabetes mellitus-induced long noncoding RNA Dnm3os regulates macrophage functions and inflammation via nuclear mechanisms. *Arterioscler Thromb. Vasc. Biol.* 38 1806–1820. 10.1161/atvbaha.117.310663 29930005 PMC6202204

[B7] Du Z. Fei T. Verhaak R. G. Su Z. Zhang Y. Brown M. (2013). Integrative genomic analyses reveal clinically relevant long noncoding RNAs in human cancer. *Nat. Struct. Mol. Biol.* 20 908–913. 10.1038/nsmb.2591 23728290 PMC3702647

[B8] Emens L. A. (2018). Breast cancer immunotherapy: facts and hopes. *Clin. Cancer Res.* 24 511–520. 10.1158/1078-0432.Ccr-16-3001 28801472 PMC5796849

[B9] Fridman W. H. Galon J. Dieu-Nosjean M. C. Cremer I. Fisson S. Damotte D. (2011). Immune infiltration in human cancer: prognostic significance and disease control. *Curr. Top. Microbiol. Immunol.* 344 1–24. 10.1007/82_2010_46 20512556

[B10] Fridman W. H. Pages F. Sautes-Fridman C. Galon J. (2012). The immune contexture in human tumours: impact on clinical outcome. *Nat. Rev. Cancer* 12 298–306. 10.1038/nrc3245 22419253

[B11] Giraldo N. A. Becht E. Remark R. Damotte D. Sautes-Fridman C. Fridman W. H. (2014). The immune contexture of primary and metastatic human tumours. *Curr. Opin. Immunol.* 27 8–15. 10.1016/j.coi.2014.01.001 24487185

[B12] Giulietti M. Occhipinti G. Principato G. Piva F. (2016). Weighted gene co-expression network analysis reveals key genes involved in pancreatic ductal adenocarcinoma development. *Cell. Oncol.* 39 379–388. 10.1007/s13402-016-0283-7 27240826 PMC13001876

[B13] Han L. Yuan Y. Zheng S. Yang Y. Li J. Edgerton M. E. (2014). The Pan-Cancer analysis of pseudogene expression reveals biologically and clinically relevant tumour subtypes. *Nat. Commun.* 5:3963. 10.1038/ncomms4963 24999802 PMC4339277

[B14] Hanzelmann S. Castelo R. Guinney J. (2013). GSVA: gene set variation analysis for microarray and RNA-seq data. *BMC Bioinformatics* 14:7. 10.1186/1471-2105-14-7 23323831 PMC3618321

[B15] Heward J. A. Lindsay M. A. (2014). Long non-coding RNAs in the regulation of the immune response. *Trends Immunol.* 35 408–419. 10.1016/j.it.2014.07.005 25113636 PMC7106471

[B16] Hu G. Gong A. Y. Wang Y. Ma S. Chen X. Chen J. (2016). LincRNA-Cox2 promotes late inflammatory gene transcription in macrophages through modulating SWI/SNF-mediated chromatin remodeling. *J. Immunol.* 196 2799–2808. 10.4049/jimmunol.1502146 26880762 PMC4779692

[B17] Hu G. Tang Q. Sharma S. Yu F. Escobar T. M. Muljo S. A. (2013). Expression and regulation of intergenic long noncoding RNAs during T cell development and differentiation. *Nat. Immunol.* 14 1190–1198. 10.1038/ni.2712 24056746 PMC3805781

[B18] Hu Q. Ye Y. Chan L. C. Li Y. Liang K. Lin A. (2019). Oncogenic lncRNA downregulates cancer cell antigen presentation and intrinsic tumor suppression. *Nat. Immunol.* 20 835–851. 10.1038/s41590-019-0400-7 31160797 PMC6619502

[B19] Huang D. Chen J. Yang L. Ouyang Q. Li J. Lao L. (2018). NKILA lncRNA promotes tumor immune evasion by sensitizing T cells to activation-induced cell death. *Nat. Immunol.* 19 1112–1125. 10.1038/s41590-018-0207-y 30224822

[B20] Huang D. W. Sherman B. T. Tan Q. Kir J. Liu D. Bryant D. (2007). DAVID Bioinformatics Resources: expanded annotation database and novel algorithms to better extract biology from large gene lists. *Nucleic Acids Res.* 35 W169–W175. 10.1093/nar/gkm415 17576678 PMC1933169

[B21] Iyer M. K. Niknafs Y. S. Malik R. Singhal U. Sahu A. Hosono Y. (2015). The landscape of long noncoding RNAs in the human transcriptome. *Nat. Genet.* 47 199–208. 10.1038/ng.3192 25599403 PMC4417758

[B22] Jansen C. S. Prokhnevska N. Master V. A. Sanda M. G. Carlisle J. W. Bilen M. A. (2019). An intra-tumoral niche maintains and differentiates stem-like CD8 T cells. *Nature* 576 465–470. 10.1038/s41586-019-1836-5 31827286 PMC7108171

[B23] Jiang C. Li X. Zhao H. Liu H. (2016). Long non-coding RNAs: potential new biomarkers for predicting tumor invasion and metastasis. *Mol. Cancer* 15:62. 10.1186/s12943-016-0545-z 27686732 PMC5043609

[B24] Jiang H. Wong W. H. (2008). SeqMap: mapping massive amount of oligonucleotides to the genome. *Bioinformatics* 24 2395–2396. 10.1093/bioinformatics/btn429 18697769 PMC2562015

[B25] Jiang X. Shapiro D. J. (2014). The immune system and inflammation in breast cancer. *Mol. Cell. Endocrinol.* 382 673–682. 10.1016/j.mce.2013.06.003 23791814 PMC4919022

[B26] Johnsen A. K. Templeton D. J. Sy M. Harding C. V. (1999). Deficiency of transporter for antigen presentation (TAP) in tumor cells allows evasion of immune surveillance and increases tumorigenesis. *J. Immunol.* 163 4224–4231. 10510359

[B27] Joshi N. S. Akama-Garren E. H. Lu Y. Lee D. Y. Chang G. P. Li A. (2015). Regulatory T cells in tumor-associated tertiary lymphoid structures suppress anti-tumor T cell responses. *Immunity* 43 579–590. 10.1016/j.immuni.2015.08.006 26341400 PMC4826619

[B28] Junttila M. R. de Sauvage F. J. (2013). Influence of tumour micro-environment heterogeneity on therapeutic response. *Nature* 501 346–354. 10.1038/nature12626 24048067

[B29] Klemm F. Joyce J. A. (2015). Microenvironmental regulation of therapeutic response in cancer. *Trends Cell Biol.* 25 198–213. 10.1016/j.tcb.2014.11.006 25540894 PMC5424264

[B30] Langfelder P. Horvath S. (2008). WGCNA: an R package for weighted correlation network analysis. *BMC Bioinformatics* 9:559. 10.1186/1471-2105-9-559 19114008 PMC2631488

[B31] Li T. Gu M. Liu P. Liu Y. Guo J. Zhang W. (2018). Abnormal expression of long noncoding RNAs in primary immune thrombocytopenia: a microarray related study. *Cell. Physiol. Biochem.* 48 618–632. 10.1159/000491890 30021206

[B32] Li X. Gruosso T. Zuo D. Omeroglu A. Meterissian S. Guiot M. C. (2019). Infiltration of CD8(+) T cells into tumor cell clusters in triple-negative breast cancer. *Proc. Natl. Acad. Sci. U.S.A.* 116 3678–3687. 10.1073/pnas.1817652116 30733298 PMC6397588

[B33] Liberzon A. Birger C. Thorvaldsdottir H. Ghandi M. Mesirov J. P. Tamayo P. (2015). The Molecular Signatures Database (MSigDB) hallmark gene set collection. *Cell Syst.* 1 417–425. 10.1016/j.cels.2015.12.004 26771021 PMC4707969

[B34] Mahmoud S. M. Paish E. C. Powe D. G. Macmillan R. D. Grainge M. J. Lee A. H. (2011). Tumor-infiltrating CD8+ lymphocytes predict clinical outcome in breast cancer. *J. Clin. Oncol.* 29 1949–1955. 10.1200/jco.2010.30.5037 21483002

[B35] Nagaraj S. Gupta K. Pisarev V. Kinarsky L. Sherman S. Kang L. (2007). Altered recognition of antigen is a mechanism of CD8+ T cell tolerance in cancer. *Nat. Med.* 13 828–835. 10.1038/nm1609 17603493 PMC2135607

[B36] Pradhan P. Qin H. Leleux J. A. Gwak D. Sakamaki I. Kwak L. W. (2014). The effect of combined IL10 siRNA and CpG ODN as pathogen-mimicking microparticles on Th1/Th2 cytokine balance in dendritic cells and protective immunity against B cell lymphoma. *Biomaterials* 35 5491–5504. 10.1016/j.biomaterials.2014.03.039 24720881 PMC4747034

[B37] Roux B. T. Heward J. A. Donnelly L. E. Jones S. W. Lindsay M. A. (2017). Catalog of differentially expressed long non-coding RNA following activation of human and mouse innate immune response. *Front. Immunol.* 8:1038. 10.3389/fimmu.2017.01038 28900427 PMC5581803

[B38] Saxena M. Bhardwaj N. (2018). Re-emergence of dendritic cell vaccines for cancer treatment. *Trends Cancer* 4 119–137. 10.1016/j.trecan.2017.12.007 29458962 PMC5823288

[B39] Schmitt A. M. Chang H. Y. (2016). Long noncoding RNAs in cancer pathways. *Cancer Cell* 29 452–463. 10.1016/j.ccell.2016.03.010 27070700 PMC4831138

[B40] Schwartz R. S. Erban J. K. (2017). Timing of metastasis in breast cancer. *N. Engl. J. Med.* 376 2486–2488. 10.1056/NEJMcibr1701388 28636861

[B41] Siegel R. L. Miller K. D. Jemal A. (2019). Cancer statistics, 2019. *CA Cancer J. Clin.* 69 7–34. 10.3322/caac.21551 30620402

[B42] Talmadge J. E. Donkor M. Scholar E. (2007). Inflammatory cell infiltration of tumors: jekyll or hyde. *Cancer Metastasis Rev.* 26 373–400. 10.1007/s10555-007-9072-0 17717638

[B43] Tamborero D. Rubio-Perez C. Muinos F. Sabarinathan R. Piulats J. M. Muntasell A. (2018). A pan-cancer landscape of interactions between solid tumors and infiltrating immune cell populations. *Clin. Cancer Res.* 24 3717–3728. 10.1158/1078-0432.Ccr-17-3509 29666300

[B44] Vesely M. D. Kershaw M. H. Schreiber R. D. Smyth M. J. (2011). Natural innate and adaptive immunity to cancer. *Annu. Rev. Immunol.* 29 235–271. 10.1146/annurev-immunol-031210-101324 21219185

[B45] Wagner J. A. Rosario M. Romee R. Berrien-Elliott M. M. Schneider S. E. Leong J. W. (2017). CD56bright NK cells exhibit potent antitumor responses following IL-15 priming. *J. Clin. Invest.* 127 4042–4058. 10.1172/jci90387 28972539 PMC5663359

[B46] Wang L. Felts S. J. Van Keulen V. P. Scheid A. D. Block M. S. Markovic S. N. (2018). Integrative genome-wide analysis of long noncoding RNAS in diverse immune cell types of melanoma patients. *Cancer Res.* 78 4411–4423. 10.1158/0008-5472.Can-18-0529 29895674 PMC6072578

[B47] Wu X. Tudoran O. M. Calin G. A. Ivan M. (2018). The many faces of long noncoding RNAs in cancer. *Antioxid. Redox Signal.* 29 922–935. 10.1089/ars.2017.7293 28793797 PMC6080117

[B48] Xu J. Cao X. (2019). Long noncoding RNAs in the metabolic control of inflammation and immune disorders. *Cell. Mol. Immunol.* 16 1–5. 10.1038/s41423-018-0042-y 29795339 PMC6318285

[B49] Yu W.-D. Wang H. He Q.-F. Xu Y. Wang X.-C. (2018). Long noncoding RNAs in cancer-immunity cycle. *J. Cell. Physiol.* 233 6518–6523. 10.1002/jcp.26568 29574911 PMC13482439

[B50] Yu Z. Zhao H. Feng X. Li H. Qiu C. Yi X. (2019). Long non-coding RNA fendrr acts as a miR-423-5p sponge to suppress the treg-mediated immune escape of hepatocellular carcinoma cells. *Mol. Ther. Nucleic Acids* 17 516–529. 10.1016/j.omtn.2019.05.027 31351327 PMC6661302

