# Supplementary figures and images for: Identification of a Six-Immune-Related Long Non-coding RNA Signature for Predicting Survival and Immune Infiltrating Status in Breast Cancer
Source: Front Genet. 2020 Jul 7;11:680. doi: 10.3389/fgene.2020.00680 (PMC7358358; doi:10.3389/fgene.2020.00680)

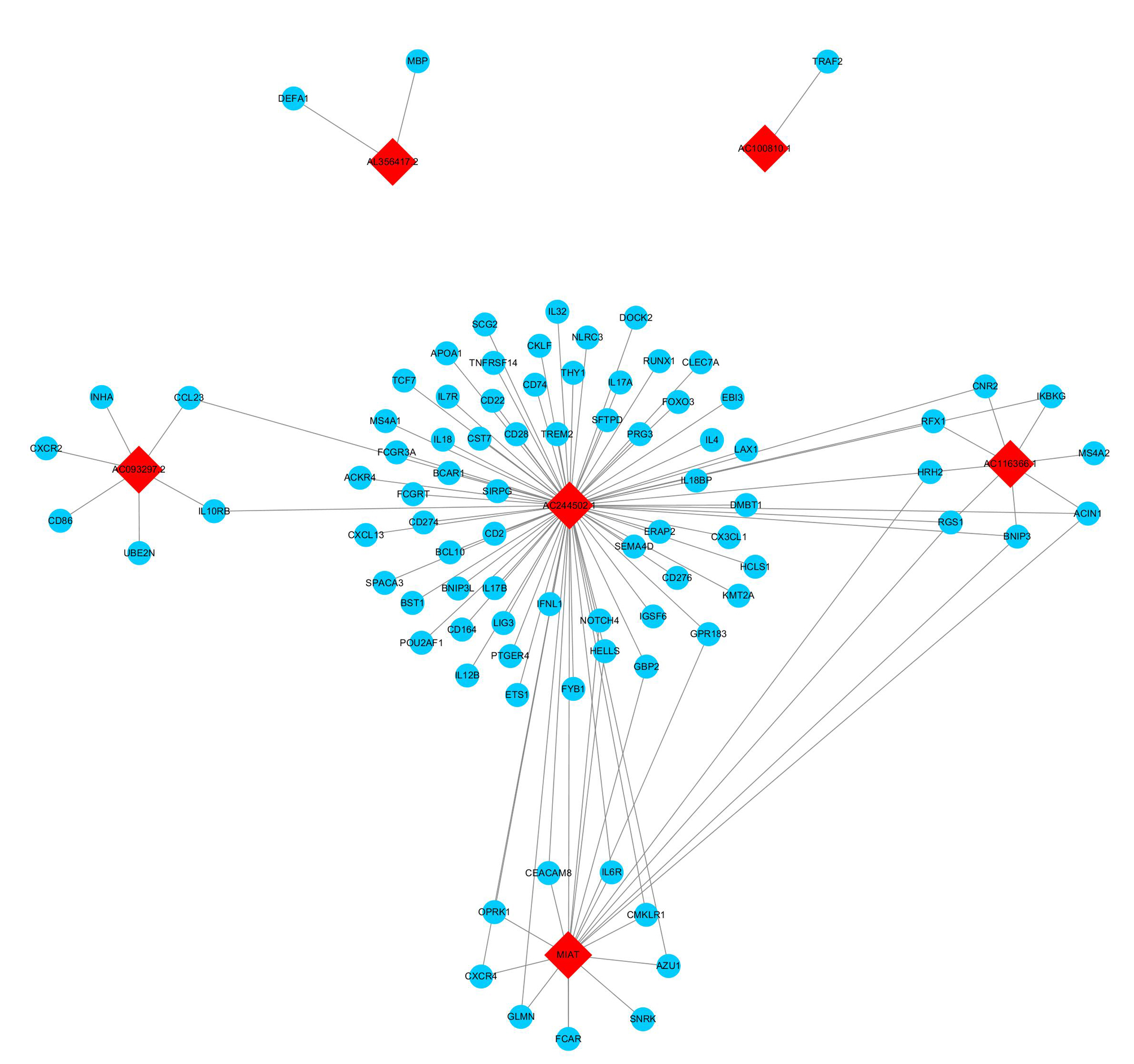

Supplement: Supplementary file 1 [file Image_1.JPEG]

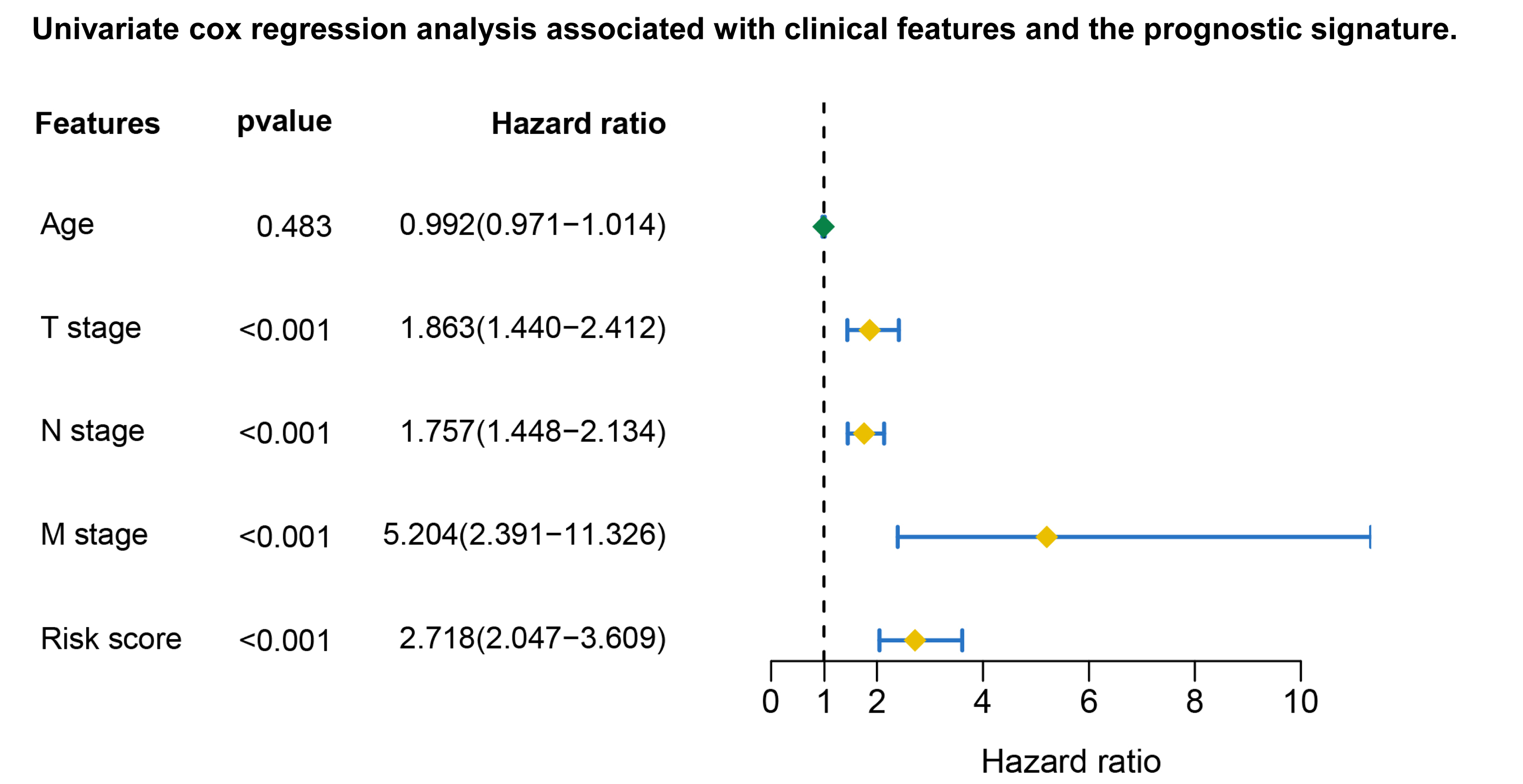

Supplement: Supplementary file 2 [file Image_2.JPEG]

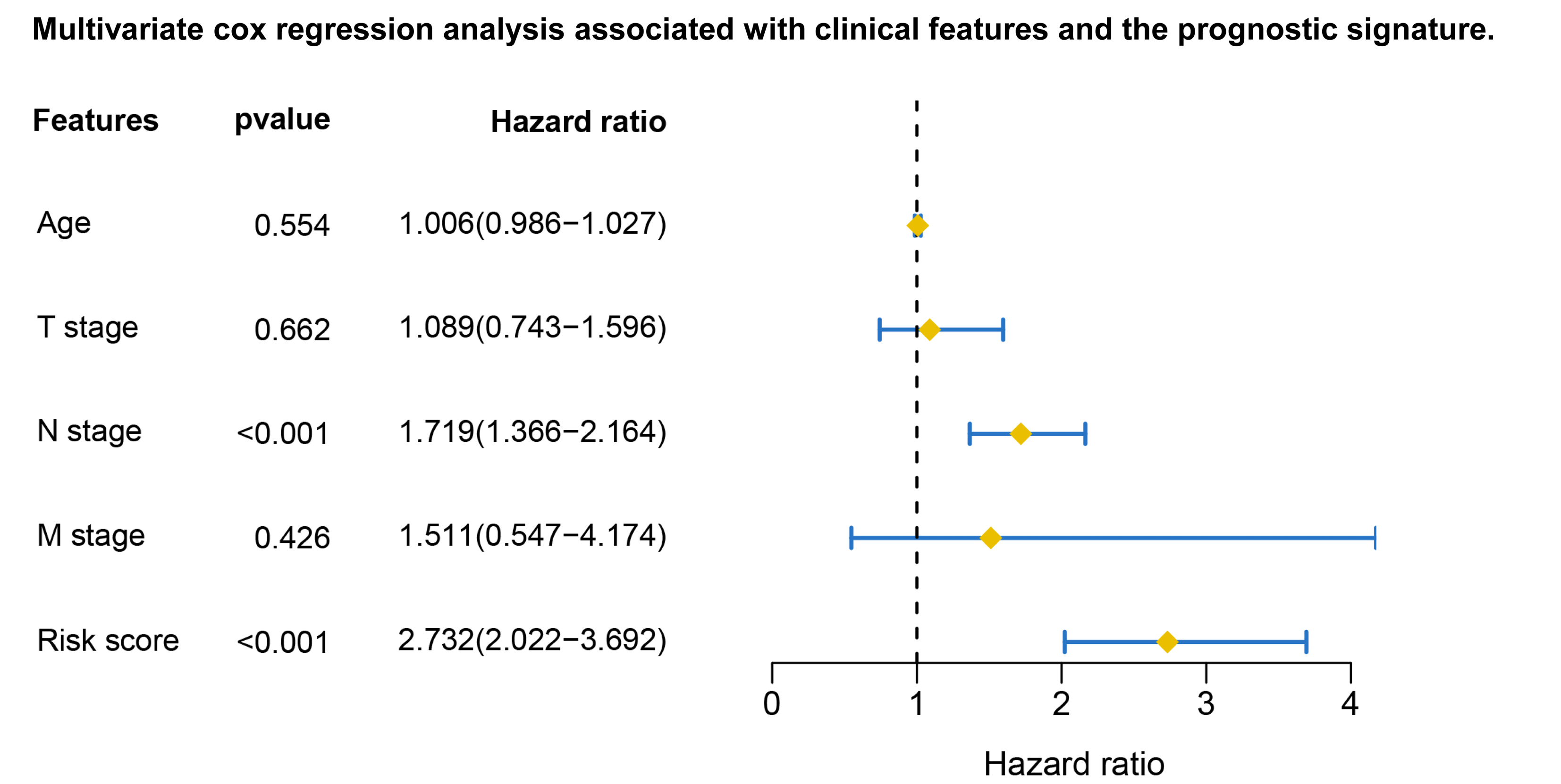

Supplement: Supplementary file 3 [file Image_3.JPEG]

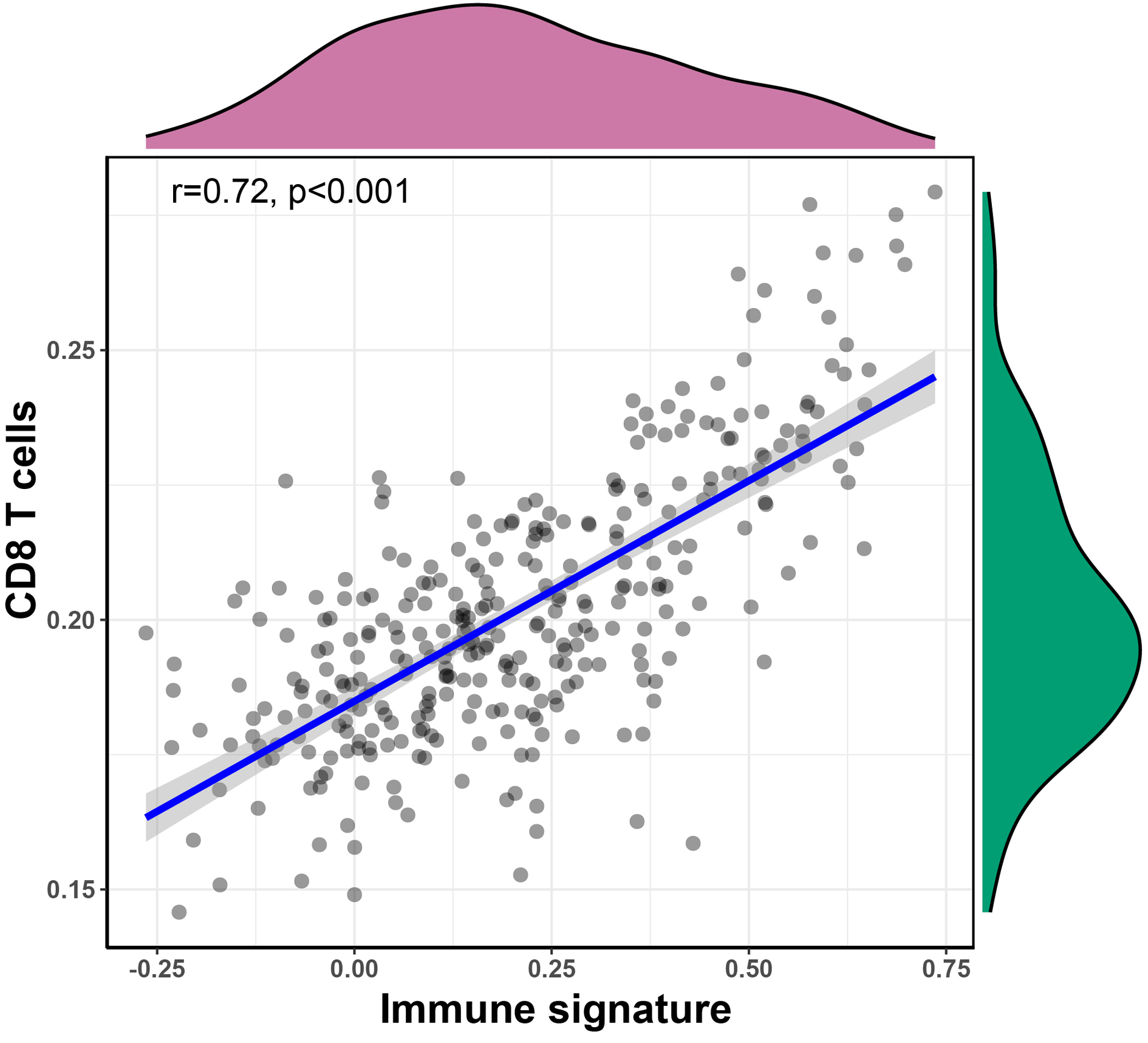

Supplement: Supplementary file 4 [file Image_4.JPEG]

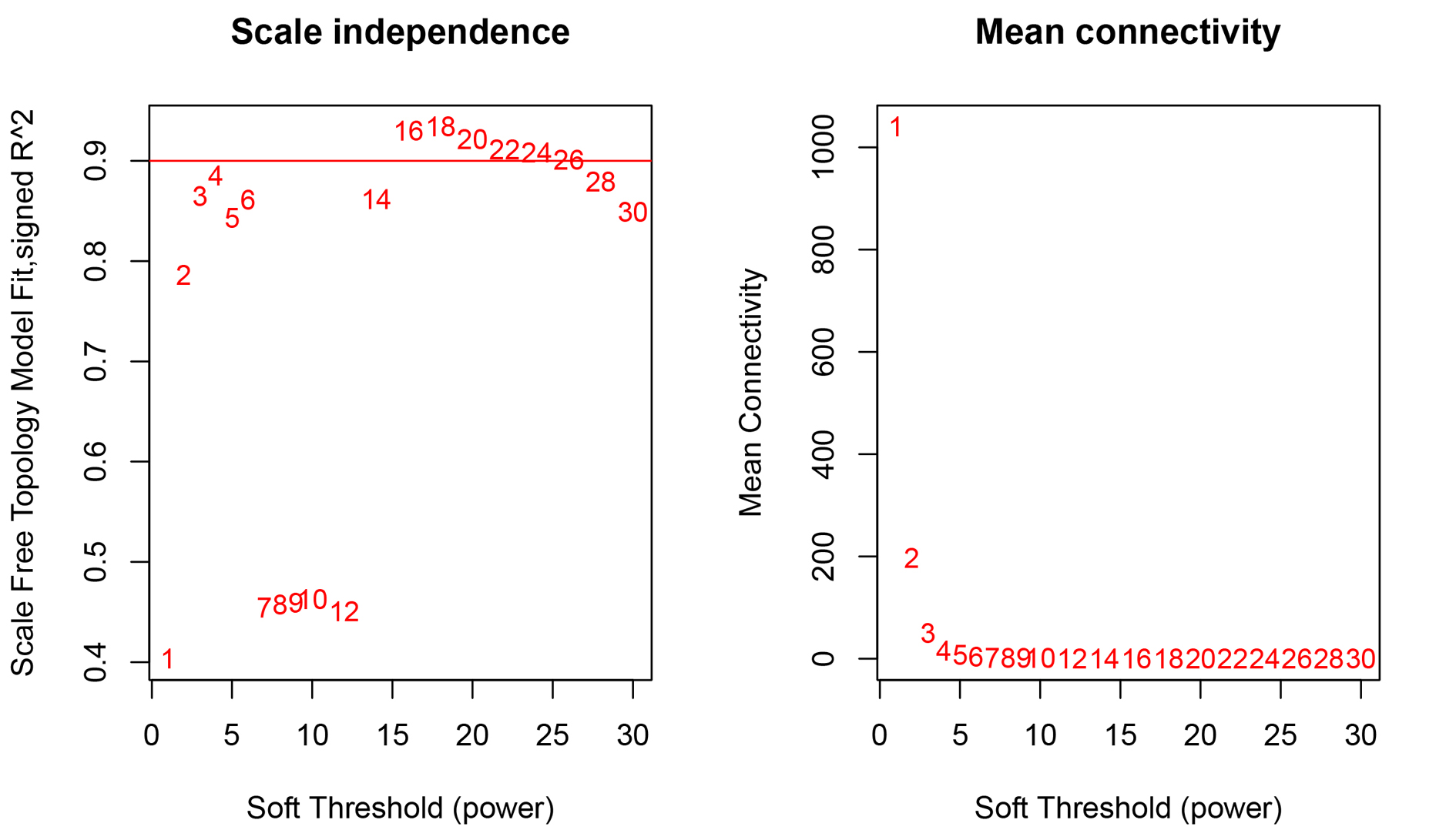

Supplement: Supplementary file 5 [file Image_5.JPEG]

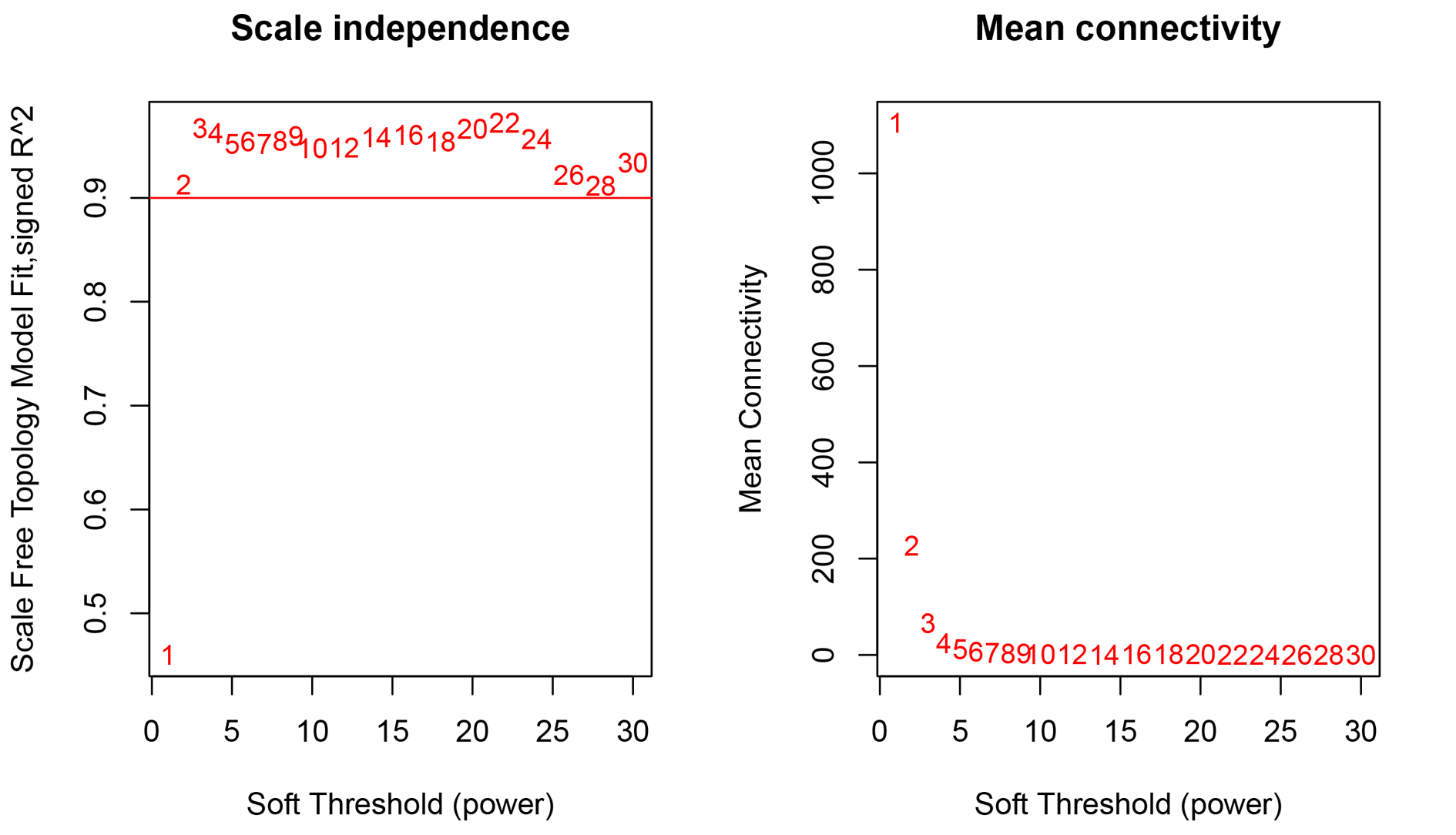

Supplement: Supplementary file 6 [file Image_6.JPEG]
